# “Pointing forehead”: a new physical sign in migraine

**DOI:** 10.1186/s40064-016-1779-3

**Published:** 2016-02-27

**Authors:** M. A. A. K. Munasinghe, Vajira Weerasinghe, M. A. S. C. Samarakoon

**Affiliations:** 1 General Hospital, Matara, Sri Lanka; 2 Department of Physiology, Faculty of Medicine, University of Peradeniya, Peradeniya, Sri Lanka

**Keywords:** Migraine, Physical sign, Cross sectional study

## Abstract

This study was designed to compare the presence of seven clinical signs in a group of patients with migraine with that of patients with non-migraine headache. Migraine is sometimes misdiagnosed. Therefore additional features are useful to improve the diagnostic accuracy of migraine. A cross sectional descriptive study was conducted in a group of 709 outpatients with headache. The physical signs were named as A–G. These were carefully observed certain gestures exhibited by patients themselves when they describe their headache. Sign A (pointing right side of the forehead) and sign B (pointing left side of the forehead) were significantly higher in patients with migraine (Sign A positive—123/339, Chi-square—15.784, p < 0.001; Sign B positive—146/339, Chi-square—20.813, p < 0.001). Sign F (keeping the head on a table) was significantly higher in patients with non-migraine headache (Sign F positive—132/370, Chi-square—12.954, p < 0.001). Sign A was more commonly associated with unilateral, severe headache which lasted for a longer period of time. However sign B was more commonly associated with unilateral, severe headache only. Sign C was significant in patients who had bilateral headache in both migraine and non-migraine groups than unilateral headache. It is concluded that pointing right or left side of forehead when the patient describes his or her headache is a characteristic sign of migraine. Keeping the head on the table during an attack of headache is not a characteristic sign of migraine.

## Background

Migraine is a common disabling primary headache disorder, characterized by recurrent attacks of moderate to severe headache, autonomic nervous system dysfunction and in some patients, an aura involving neurological symptoms.

Migraine affects 11 % of adults (Stovner et al. 2007) and 6.2 % of children (Mavromichalis et al. 2007) worldwide, is more common in women than men (Lipton et al. 2007) and is most prevalent in the third and fourth decades of life. Approximately one-third of individuals with migraine experience neurological aura symptoms before headache onset (migraine with aura), usually consisting of transient visual, and also sensory, aphasic, or motor disturbances (Ferrari 1998). In migraine without aura (previously known as common migraine), attacks are usually associated with nausea, vomiting, or sensitivity to light, sound, or movement. When untreated, these attacks typically last 4–72 h.

Migraine is often misdiagnosed as sinus headache (Schreiber et al. 2004) or tension type headache. Therefore additional features are useful to improve the diagnostic accuracy of migraine. A previous study has been done in patients with hemiplegic migraine to show that the digiti quinti sign is an indication of mild hemiparesis (Vincent 2009). Another study has shown that an after image following fundoscopy is more common in patients with migraine (De Silva 2001). Apart from these studies there has not been any other sign previously described which correlates with migraine.

It is our observation that certain gestures or pointing signs are often present in these patients. We have included 7 physical signs which we have noted in patients who were thought to have migraine. The method of elicitation and detailed description of physical signs will be explained under methodology.

Therefore the present study was done to investigate the importance of the above signs in order to improve the clinical diagnosis of migraine.

## Objective

Objective of this study is to compare the presence of seven clinical signs in a group of migraine patients with that of patients with non-migraine headache.

## Methods

This is a cross sectional descriptive study with experimenter blinding which was conducted in the General Hospital, Matara, Medihouse private hospital, Matara and suburban peripheral hospitals using outpatients who presented with headache attending to medical and neurological clinics and OPD.

Study was conducted in 709 patients with headache including males and females between age groups of 15–45 for a period of 1 year. Patients with migraine (test sample) and patients with non-migraine headache (control sample) were diagnosed based on the International Classification of Headache Disorders (Headache Classification Committee of the International Headache Society 2004).

Based on the International Classification of Headache Disorders (Headache Classification Committee of the International Headache Society 2004) we have formulated the following exclusion criteria.

Exclusion criteria

1. headache attributed with head and/or neck trauma
2. headache attributed to cranial or cervical vascular disorder
  - ischaemic stroke or transient ischaemic attack
  - Non-traumatic intracranial haemorrhage
  - Unruptured vascular malformation
  - Arteritis
  - Carotid or vertebral artery pain
  - Cerebral venous thrombosis
  - Other intracranial vascular disorder
3. Headache attributed to non-vascular intracranial disorder
  - Increased cerebrospinal fluid pressure
  - Decreased cerebrospinal fluid pressure
  - Epilepsy
  - Non-infectious inflammatory disease
  - intrathecal injection
  - Intracranial neoplasm
  - Other non-vascular intracranial disorders
4. Headache attributed to a substance or its withdrawal
5. Headache attributed to infection
  - Intracranial infection- bacterial/viral meningitis, encephalitis, brain abscess
  - Systemic infection
6. Headaches attributed to disorder of homoeostasis
  - Arterial hypertension
  - Hypoxia and/or hypercapnoea
  - Dialysis headache
  - Hypothyroidism
  - Fasting
7. Headache or facial pain attributed to disorder of cranium, neck, eyes, ears, nose, sinuses, teeth, mouth or other cranial or facial structures
8. Headache attributed to psychiatric disorder

Each patient was given an interviewer administered questionnaire (e-Questionnaire) to collect information about the headache. Trained assistant medical investigators with a medical background (pre-intern medical officers) were used to administer the questionnaire. Each investigator was trained by the principal investigators using the mock scenario referred to figures below shown. All assistant investigators were blinded with regard to the physical signs. From time to time assistant investigators were supervised personally by the principal investigators whether they were following the correct methodology in which they were trained.

Before the administration of questionnaire informed verbal consent was obtained. The physical signs were named from A–G. It is important to note that we didn’t elicit the physical sign as such. Instead patients themselves exhibited certain gestures spontaneously when they describe their headache which we have named as “signs” A, B, C, D and E. “Signs” F and G were noted by asking the patient about the presence of those signs during an attack. Ethical clearance for the study was obtained from the Ethical Committee, Faculty of Medicine, University of Peradeniya, Peradeniya, Sri Lanka.

- A: Pointing right side of forehead
- B: Pointing left side of forehead
- C: Pointing both sides of forehead
- D: Wrinkling the forehead
- E: Touching the forehead
- F: Keeping the head on the table (Keeping the head on the arm which is on the table)
- G: Lying on bed

**Fig. 1:**
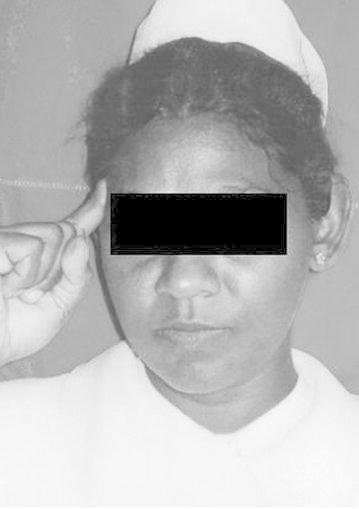
Sign A: Pointing *right side* of forehead

**Fig. 2:**
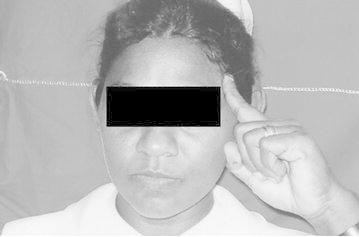
Sign B: Pointing *left side* of forehead

**Fig. 3:**
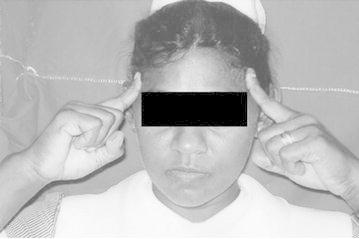
Sign C: Pointing *both sides* of forehead

**Fig. 4:**
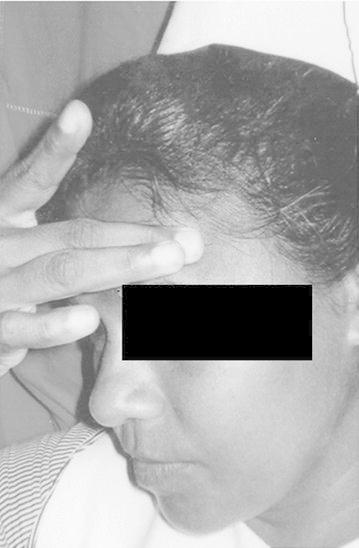
Sign D: Wrinkling the forehead

**Fig. 5:**
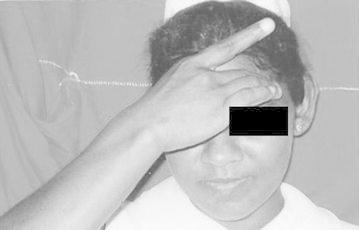
Sign E: Touching the forehead

**Fig. 6:**
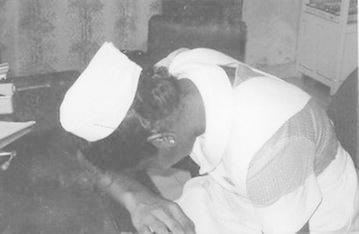
Sign F: Keeping the head on the table (keeping the head on the arm which is on the table)

**Fig. 7:**
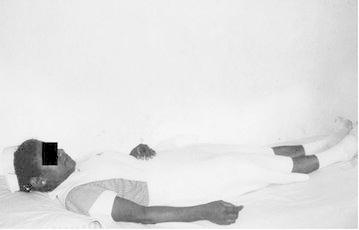
Sign G: Lying on bed

These signs are shown in Figs. 1, 2, 3, 4, 5, 6 and 7.

Results were analyzed using SPSS 10.

## Results

There were 339 patients with migraine (Group 1) and 370 patients with non-migraine headache (Group 2). Presence of signs A, B, C, D, E, F and G were compared between Group 1 and Group 2 and the results are presented in Table 1.

**Table 1:** Number (%) of patients with the presence of signs A, B, C, D, E, F and G between the two groups

	Group 1	Group 2	Chi-square	Significance (p value)
A	123/339 (36.3 %)	84/370 (22.7 %)	15.784	<0.001
B	146/339 (43.1 %)	99/370 (26.8 %)	20.813	<0.001
C	14/339 (4.1 %)	19/370 (5.1 %)	0.403	0.594
D	144/339 (42.5 %)	143/370 (38.6 %)	1.077	0.320
E	68/339 (20.1 %)	93/370 (25.1 %)	2.597	0.127
F	79/339 (23.3 %)	132/370 (35.7 %)	12.954	<0.001
G	162/339 (47.8 %)	200/370 (54.1 %)	2.780	0.099

Statistical significance is given as p values

Signs A and B were significantly higher in patients with migraine than with non-migraine headache.

There was no significant difference between the two groups in signs C, D, E and G. But the sign F was significantly higher in patients with non-migraine headache.

As signs A and B were significantly higher in patients with migraine, association of these 3 signs with factors such as how long the patient has had migraine, duration of one attack of migraine, location (unilateral/bilateral) of the headache, severity of the headache, presence of aura symptoms, duration of aura, interval between aura were analyzed in patients with migraine.

## How long the patient had migraine (months)

In the migraine group, the total duration of migraine was 57.38 ± 6.79 (mean and SE) months when the sign A was positive and 61.23 ± 5.57 months when the sign A was negative. This difference was not statistically significant (t value: 0.42, p value: 0.669).

In the migraine group, the total duration of migraine was 51.05 ± 6.98 (mean and SE) months when the sign B was positive and 66.48 ± 5.41 months when the sign B was negative. This difference was not statistically significant (t value: 1.775, p value: 0.077).

## Duration of one attack in hours

Duration of an attack between the patients with the sign positive and negative are shown in Table 2.

**Table 2:** Duration of one attack (in hours, mean and std. error) between those with sign positive and with sign negative shown in the table

	Sign positive group	Sign negative group	t value	Significance (p value)
Sign A	26.37 (±2.36)	21.09 (±1.44)	2.026	0.044
Sign B	22.14 (±1.65)	23.65 (±1.83)	0.593	0.553

As shown in the Table 2 sign A was detected more in patients who has had one attack for a prolong duration with a t value of 2.02 and p value less than 0.05 making the presence of sign A statistically significant. However there was no statistical significance of the association of sign B with the duration of an attack.

## Location of the headache

Among migraine patients who have had sign A positive, 86.2 % had unilateral headache while 13.8 % had bilateral headache whereas in migraine patients who have had sign A negative only 52.3 % had unilateral headache. This difference was statistically significant (Chi-square value: 39.302, p value: <0.001).

Among migraine patients who have had sign B positive, 80.1 % had unilateral headache while 19.9 % had bilateral headache whereas in migraine patients who have had sign B negative only 52.8 % had unilateral headache. This difference was statistically significant (Chi-square value: 27.065, p value: <0.001).

As sign A and B were found in patients with unilateral migraine with a high statistical significance, association of sign C was checked with the location.

Presence of sign C was highly significant in patients who had bilateral headache in both migraine (14/339) and non-migraine (19/370) groups than unilateral headache (migraine-1/339 and nonmigraine-2/370) with a p value less than 0.05.

Migraine group—Chi-square value: 21.083, p value: <0.001

Non-migraine group—Chi-square value: 5.128, p value: 0.025

## Severity of the headache

Sign A was found more commonly in migraine patients with severe headache (95/123, 77.2 %) than moderate headache (28/123, 22.8 %) with a high statistical significance (Chi-square value: 4.210, p value: 0.047).

Sign B was also found more commonly with severe headache (91/146, 62.3 %) than moderate headache (55/146, 37.7 %) with a high statistical significance (Chi-square value: 8.236, p value: 0.006) in the migraine group.

There was no statistical significance of signs A or B with the aura symptoms except in the case of aphasia. Both sign A and B were detected in migraine patients who have had aphasia as an aura symptom with a p value less than 0.05. There was no statistical significance of signs A and B with the duration of Aura or the interval between auras.

## Discussion

Migraine is a clinical diagnosis. This study describes important clinical signs which would improve the diagnostic accuracy of migraine. It was conduced in a group of 709 patients presented with headache.

Pointing right or left side of forehead (signs A and B) was statistically significantly associated with the migraine group when the patient describes his or her headache. Furthermore, sign A was more commonly associated with unilateral, severe headache which lasted for a longer period of time. However sign B was more commonly associated with unilateral, severe headache only. Sign C (Pointing both sides of forehead) was more common in patients who had bilateral headache in both migraine and non-migraine groups. Keeping the head on the table (Sign F) was significantly associated in patients with non-migraine headache.

These signs have not been described in previous studies apart from the after image following fundoscopy (De Silva 2001) and the digiti quinti sign (Vincent 2009) which has limitations because it can be only used in hemiplegic migraine.

These results show that pointing right side or left side of forehead as a gesture is a sign of migraine which is common mainly in unilateral severe headache. Although both sign A and B should be having the equal significance when considering in the context of the duration of the attack, according to the statistical analysis only sign A is significant. Although it is difficult to explain this paradox, handedness and the cerebral dominance may be a factor. In our study handedness was not investigated.

When considering the relationship of Sign A and B (here in after referred to as AM sign of migraine [AM-Aruna Munasinghe] for the sake of easiness) taken together with its strong statistical significance with the severity, it is reasonable to propose that so called AM sign can be considered as a measure of severity as well.

It is still interesting to find that both sign A and B (AM sign) is positive with aphasia as an aura. We are not in a position to explain this phenomenon. As we have mentioned above it may be related to handedness of the people. (Whether they are right handed or left handed.)

This study was performed by using an interviewer administered questionnaire. Although all the interviewers were properly trained by the principal investigators it is impossible to believe that they will administer the questionnaire with the same accuracy as there is always a bias factor. It is important to note that person who analyzed the data is completely blind with regard to the physical signs.

Although we have done a detailed literature review we could not find any comparable or similar study. Hence this study is a unique one. For the sake of understanding this can be compared with the pointing sign in appendicitis. However in appendicitis the sign is elicited by the examiner whereas our pointing sign (AM sign) is not elicited in the same manner. Instead the sign itself is shown by the patient as a gesture. The examiner only observes the gesture and interprets it as a sign. Corrigan’s sign in aortic incompetence is a reasonable comparison to so called ‘AM’ sign. The importance of this sign lies in the fact that it shows a very strong statistical significance in the clinical setting. Therefore the signs we have described will be very useful in making a positive diagnosis of migraine which is often diagnosed by exclusion by many clinicians, despite international headache society criteria. It is important to stress that sign F (arbitrarily named as SM sign) has a strong negative predictive value thus helping ruling out migraine in clinical diagnosis of headache.

There may be cultural/regional variations of patients with headache, communicate with their doctor. If pointing sign could be confirmed in a study in another country this would support the universality of the finding and the generalization of the conclusions.

In conclusion pointing right or left side of forehead when the patient describes his or her headache is a characteristic sign of migraine. Keeping the head on the table during an attack of headache is not a characteristic sign of migraine.
